# Barriers to and Recommendations for Equitable Access to Healthcare for Migrants and Refugees in Aotearoa, New Zealand: An Integrative Review

**DOI:** 10.1007/s10903-023-01528-8

**Published:** 2023-09-04

**Authors:** Blessing Kanengoni-Nyatara, Katie Watson, Carolina Galindo, Nadia A. Charania, Charles Mpofu, Eleanor Holroyd

**Affiliations:** 1 AUT Migrant and Refugee Health Research Centre, Faculty of Health and Environmental Sciences, School of Public Health and Interdisciplinary Studies, Auckland University of Technology, 90 Akoranga Drive, Northcote, Auckland, 0627 New Zealand; 2 Hato Hone St John, 600 Great South Road, Ellerslie, Auckland, 1051 New Zealand; 3 AUT Migrant and Refugee Health Research Centre, School of Public Health and Interdisciplinary Studies, Auckland University of Technology, 90 Akoranga Drive, Northcote, Auckland, 0627 New Zealand

**Keywords:** Equity, Access, Migrants, Refugees, Healthcare, Barriers, Aotearoa New Zealand, Review

## Abstract

The health system in Aotearoa New Zealand is predicated on equity in access to health services as a fundamental objective yet barriers to equitable access for migrant and refugees continue to exist. There is a paucity of studies that synthesise the experiences and realities of migrants, refugees and healthcare providers that hinder access to healthcare and provide recommendations to improve services. This review synthesised these barriers and recommendations, with an aim to improve equitable access to healthcare to migrants and refugees. An integrative review of 13 peer-reviewed research studies from EBSCOhost research databases published between January 2016 and September 2022. Studies included: (i) related to Aotearoa; (ii) had a focus on equitable delivery of healthcare to migrants and refugees; and (iii) had a full English text available. The PRISMA framework guided the reporting of the review. The findings were thematically analysed and presented using a narrative empirical synthesis. The findings were organised into three broad themes: attitudinal barriers, structural barriers, and recommendations. Attitudinal barriers included the lack of culturally competent healthcare providers, discrimination by healthcare providers, and personal, social, and cultural attributes. Structural barriers referred to policies and frameworks that regulated the accessibility of health services such as the cost of healthcare, accessibility and acceptability of interpreter services, length of allocated appointments and long waiting times for an appointment, difficulties navigating the health system, and logistical barriers. Recommendations focused on promoting a sense of belonging, enabling a whole-of-society approach that brings together all sectors involved in providing health care for collective impact, and advocating for government policies to create a system that addresses the core health service access needs. This review provides rich context-specific findings on the barriers to equitable access to healthcare and proposed interventions to enhance equitable health outcomes for migrants and refugees in Aotearoa. The review contributes to relevant policy decisions and has practical implications to build responsive health systems which are inclusive, equitable and best address the health needs of populations from diverse cultural backgrounds.

## Introduction

Inequities in access to health care are seen when there are systematic differences in related factors, such as socio-economic conditions, migrant status, or ethnicity, rather than need [1]. A recent systematic review identified several barriers in Organization for Economic Co-Operation and Development (OECD) countries that hinder migrants’ and refugees’ access to health care, including legal status, linguistic and cultural issues, health providers not being provided with past health records, lack of assistance or provision of information on navigating the care and support system, lack of coordination between healthcare providers, and poor organisation and quality of healthcare services [2]. Of note is that during the COVID-19 pandemic, temporary migrant workers, especially migrant farm workers and international students in Aotearoa New Zealand, Canada and Australia, remained excluded from health services and social protection [3]. Moreover, research in Aotearoa notes unique and specific barriers in accessing healthcare between migrant and former refugee populations [4, 5].

The distinction between migrants and former refugees is therefore important when discussing variations in access. The International Organisation for Migration describes a migrant as "an umbrella term, not defined under international law, reflecting the common lay understanding of a person who moves away from his or her place of usual residence, whether within a country or across an international border, temporarily or permanently, and for a variety of reasons." p.132 [6]. According to the 1951 Refugee Convention relating to the Status of Refugees and its 1967 Protocol, a refugee is a person who, “owing to a well-founded fear of persecution for reasons of race, religion, nationality, membership of a particular social group or political opinions, is outside the country of his nationality and is unable or, owing to such fear, is unwilling to avail himself of the protection of that country” [7]. Environmental migration is now a part of the migration discourse with terms such as “climate migration” and “disaster displacement” describing the multitude of ways in which people move from one place to another [8]. Prior to the COVID-19 pandemic, the United Nations estimated that there were 281 million international migrants in 2020, which equates to 3.6% of the global population [9]. Recent estimates report that 108.4 million people have been forcibly displaced and of these, 35.3 million are refugees and 5.4 million are asylum seekers [10]. The large increase in displaced persons, migrants and refugees seen in 2014–2016 brought a new urgency to global efforts to achieve equity in access to health services [11].

Aotearoa has witnessed several recent migration pathways driven by changes to migration polices that have shaped its demographic landscape, these include the Skilled Migrant Category (2009) for skilled workers to obtain residency based on their qualifications, work experience, and English language proficiency, the Entrepreneur Work Visa (2012) to encourage migrant entrepreneurs to establish businesses, an expansion of the Refugee Quota Program which saw the doubling of the annual refugee quota by 2020, and the community organisation refugee sponsorship (CORS) pilot programme in 2019 that allows community groups to sponsor and support refugee families. These polices have resulted in an influx of skilled migrants, particularly in sectors such as information technology, healthcare, and construction. In addition, the country has seen increases in international students choosing to study in educational institutions thereby pursuing pathways to gain work experience and opportunities for long-term settlement [12]. Furthermore, Aotearoa is also experiencing a slow but steady rise in refugees, evacuees and asylum seekers, with individuals seeking safety and security from conflict and persecution in their home countries [13]. It is important to note that the temporary border restrictions associated with the ongoing COVID-19 pandemic has limited the entry of migrants and refugees into the country.

In Aotearoa, the refugee quota program allows up to 1500 refugees to be resettled annually, up from the established annual global quota system of 750 refugees in 1997 [14]. Quota refugees are granted permanent residence upon arrival and offered additional off-shore and on-shore health and orientation support [15]. This is in addition to resettling asylum seekers under the refugee and protection programme (known as convention refugees if their claim is successful), family members via the family reunification scheme (up to 600 annually), and refugees under the CORS programme [16]. Those resettled via the refugee family support category [17] and CORS [18] are granted permanent residence upon arrival, while convention refugees are eligible to apply for permanent residence [19]. In 2016/17, other visa categories witnessed a net increase of 72,300 permanent and long-term migrants, a 4.7% increase over the 2015/16 figures and the fifth consecutive year in which migration increased, recording the highest net gain ever [20]. This increasingly multicultural society demonstrates the challenge of delivering culturally responsive and appropriate services to migrant and refugee communities, and the need for cultural understanding by healthcare providers [21].

Most countries of resettlement have primary health care as the initial point of access into the health system [22]. In Aotearoa, the healthcare system is a universal, tax-funded national health service with no-fault accident coverage [23]. Individuals must meet the eligibility criteria to be considered for publicly funded (i.e., free or subsidised) health and disability services [24], which can have implications for healthcare access among migrants and refugees. Generally, citizens and permanent residents of Aotearoa, those on valid interim visas, and those on a work visa that entitles them to stay in the country for two years or more are eligible [24]. Those with refugee or protection status (asylum seekers) are also eligible for subsidised health care with proof of their status [25]. The healthcare system is divided into three main service components: (i) public health services that provide and shape policies to promote areas that make a difference to lifelong health, such as immunisation and the management of outbreaks of infectious diseases, like COVID-19; (ii) primary healthcare which is the entry level into the health system and includes a broad range of activities and services from health promotion and prevention to the treatment and management of acute and chronic conditions; and (iii) secondary health care that is often based in a hospital setting and unlike primary care, requires a referral [23].

However, the national health system continues to struggle with access inequities for all population groups [23], with pronounced barriers for effective health care for migrant and refugees [26]. There is a paucity of studies in Aotearoa that synthesise the experiences and realities of migrant and refugee groups when accessing healthcare and the healthcare providers who deliver services to these populations. The presented review synthesises the evidence on barriers to accessing healthcare services and where present, propose interventions to improve services in various healthcare settings for migrants and refugees. This review has implications for building responsive health systems that provide equitable access and best address the health needs of populations from diverse cultural backgrounds [27].

## Method

An integrative review was undertaken to answer the research questions as this approach allowed for the inclusion of a variety of literature about the experiences from the perspectives of migrants, refugees and healthcare providers, in addition to capturing recommendations for providing equitable access to healthcare [28]. The following research questions guided the presented review:

1. What are the barriers to equitable access to healthcare services for migrant and refugee communities in Aotearoa?
2. What are the recommendations to support equitable access to healthcare services for migrant and refugee communities in Aotearoa?

### Inclusion/Exclusion Criteria

Eligible studies were those published between January 2016 to September 2022 to mirror the adoption of the 2030 Sustainable Development Agenda in 2015 [29]. Only studies written in English, as all the multi-disciplinary researchers have English as the common language, were included. Included studies: (i) related to Aotearoa; (ii) had a focus on equitable delivery of healthcare for migrants and refugees; and (iii) had a full English text available. Studies on Pacific migrants were excluded as this group requires in-depth consideration in light of their historical and current context and thus warrants a separate review. Also, as we were not able to discern the migration background of authors, criteria pertaining to the authorship was not included as part of the inclusion/exclusion criteria. Grey literature, such as government documents, was excluded as the focus was on empirical, peer-reviewed studies.

### Search Strategy

Nine EBSCOhost research databases were searched, including MEDLINE, Australia/New Zealand Reference Centre, Dentistry and Oral Sciences, SocINDEX, Business Source Complete, Communication and Mass Media Complete, SPORTDiscuss, Humanities International Index and CINAHL complete. Table 1 outlines variations of common terms used in global research studies that guided the search and Table 2 outlines additional search terms focused on the COVID-19 pandemic.

**Table 1:** Search terms used to search for studies published in English from January 2016 to September 2022

Migrant* or refugee* or "undocumented migrant*" or "ethnic minorit*" or "first generation migrant*" or "second generation migrant*" or immigrant or "newcomer*"
**AND** Healthcare or "health care" or "general practitioner " or doctor* "health system" or "health service*" or "health professional*" or "primary health care*" or "health promotion" or "health behaviour*" or " or "health program*" or "health policy*" or "health project*" or ambulance
**AND** Barrier* or facilitator* or access* or equit* or inequt* or hinder* or enable* or unsuccess* or success*
**AND** "New Zealand" or NZ or "Aotearoa NZ"

**Table 2:** Search terms used to search for studies published in English from January 2016 to September 2022 with a focus on the COVID-19 pandemic

Migrant* or refugee* or "undocumented migrant*" or "ethnic minorit*" or "first generation migrant*" or "second generation migrant*" or immigrant or "newcomer*"
AND Healthcare or "health care" or "general practitioner " or doctor* "health system" or "health service*" or "health professional*" or "primary health care*" or "health promotion" or "health behaviour*" or " or "health program*" or "health policy*" or "health project*" or ambulance
AND Covid-19 or coronavirus or 2019-nCoV or SARS-CoV-2 or CoV-19
AND Barrier* or facilitator* or access* or equit* or inequt* or hinder* or enable* or unsuccess* or success*
AND "New Zealand" or NZ or "Aotearoa NZ"

### Data Extraction

**Fig. 1:**
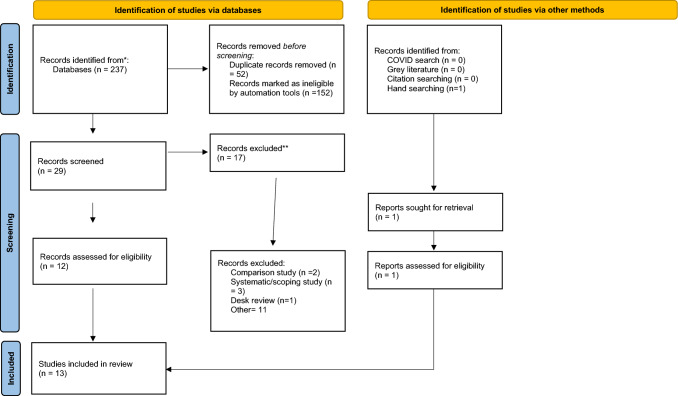
PRISMA flowchart showing the studies identified and the process of inclusion and elimination

The searches were conducted by the lead author (BKN). After removing duplicates, the titles and abstracts of the remaining studies were screened to assess if they addressed the research questions and met the inclusion criteria; any irrelevant studies were excluded. The process of title and abstract screening was undertaken independently by three authors (BKN, KW, CG). The studies were retained by consensus if there were any disagreements and then subjected to a full-text review. Reference lists of the selected studies were searched for additional references. Two authors (BKN, EH) independently reviewed the full-text studies for relevance and inclusion. Members of the research team (BKN, KW, CG, NC, CM, EH) met regularly to review progress, discuss any discrepancies about eligibility and decide on the final studies for inclusion and identified themes. The process of selection was guided by the Preferred Reporting Items for Systematic Reviews and Meta-Analyses (PRISMA) flowchart (Fig. 1).

### Data Analysis and Synthesis

The findings were thematically analysed. BKN, KW, CG, and EH created the themes using vote counting to identify the frequency with which themes appeared in the included studies. The vote count for each theme comprised the number of studies mentioning either the theme itself or a subordinate theme [30, 31]. Some new themes were created, and others were subsumed within existing themes and upon discussion and agreement, were given less prominence, or deleted. On completion of the thematic analysis and vote counting, a narrative synthesis approach was used to combine and connect the findings of the individual studies and identify relationships between them for a collective broader perspective [32].

## Results

Of the 237 studies identified, 13 satisfied the inclusion criteria and were included in the review.

* The records removed by an automation tool (n = 152) were studies conducted outside Aotearoa.

### Characteristic of the Included Studies

Table 3 outlines the key characteristics, findings, and recommendations of the included studies. Most studies (n = 12) were qualitative, and one study used a mixed methodology. Although the review focused on both migrants and refugees, the majority of studies (n = 8) focused on refugee populations [26, 33–39], one article focused on ‘refugee-like migrants’ (legal migrants with refugee-like backgrounds, e.g., migrant family members with refugee backgrounds) [5], one article was on “new settlers” [40], and three were on migrants [41–43]. All study participants came from low and middle-income or non-English speaking countries.

**Table 3:** The selected studies were summarised in a chart to include authors, research question, and sample size, type of study, findings and recommendation

Author & article	Aim/Research question	Type of study	Sampling approach	Sampling size	Findings	Recommendation
Kennedy, Kim, Moran, and McKinlay (2021) Qualitative experiences of primary health care and social care professionals with refugee-like migrants and former quota refugees in New Zealand. *Australian journal of primary health, 27(5),* 391–396. 10.1071/PY20285	To examine the experiences of primary care professionals, finding key themes for successful care	Exploratory qualitative study	Purposive	12 healthcare workers	Similarities exists between refugee-like migrants and former quota refugees Barriers still present affecting the delivery of core health and support services	Migrants, especially family members of former refugees, may have similar health and social experiences to former refugees. Health and social care professionals should take these experiences into account when planning and providing care
Richard, Richardson, Jaye, and Stokes (2019) Providing care to refugees through mainstream general practice in the southern health region of New Zealand: a qualitative study of primary healthcare professionals’ perspectives. *BMJ Open, 9*(12), e034323. 10.1136/bmjopen-2019-034323	To explore the perspectives of primary healthcare (PHC) professionals providing care to refugees through mainstream general practice	Qualitative exploratory design with semi-structured interviews	Purposive	Nine general practitioners and six practice nurses enrolled in the Dunedin Refugee Resettlement Programme, in New Zealand	Building meaningful relational connections involved acknowledging refugees’ journeys by getting to know them as people Participants encountered challenges in providing care to refugees with respect to time-limited consultations, variable use of interpreter services, fragmentation of care between agencies and need for improved health infrastructure to ensure a fluid interface between PHC, secondary care and community support services The current business model of NZ general practice was perceived to interfere with value-driven care and discouraged tailoring of care to specific patient groups	Health care professional to advocate for people from refugee backgrounds to influence policy makers to recognise the unique individual, social, cultural and historical factors that affect their health and promote a culture of acceptance that celebrates diversity Mainstreaming of gender in the delivery of these services for culturally appropriate practice, to facilitate relationship building and trust Health service providers to collaborate with non-government organisations that work with migrants and refugees to include establishing an interprofessional team within and across practises, sharing systems and information and investing in skill development and teamwork between practices
Shrestha-Ranjit, Patterson, Manias, Payne, and Koziol-McLain (2020) Accessibility and acceptability of health promotion services in New Zealand for minority refugee women. *Health promotion international*, *35*(6), 1484–1494. 10.1093/heapro/daaa010	To examine the accessibility and acceptability of health promotion services for Bhutanese refugee women who resettled in New Zealand	Qualitative	Purposive	32 Bhutanese women and eight Bhutanese men; 12 individual interviews with health professionals 18 to 82 years and men’s from 26 to 55 years	Bhutanese women were missing some essential health promotion services, such as antenatal education sessions mainly due to language and cultural barriers	To develop health promotion resources in the Nepali language; and to deliver the health promotion sessions by culturally and linguistically competent providers
Field, McClunie-Trust, Kearney, and Jeffcoat (2020) Language and communication: A vital component of Health for people with Refugee backgrounds. *KaiTiaki Nursing Research, 29 (3)*. ISSN 1179/772x (In Press)	To explore transdisciplinary understandings of the implications of language development for the health and wellbeing of people who have come as refugees to Aotearoa New Zealand To analyse the health implications of an emergent data set from a primary research study with learners who were refugees	Qualitative	Purposive	60 adults of refugee backgrounds from Somalia, Cambodia, Colombia, Pakistan, Democratic Republic of Congo and Afghanistan. 18 to 64 years old, predominantly female	Key themes developed from the secondary analysis were, complexity of life experience, challenges to living and learning, family responsibilities, challenges to ‘peace of mind’ and mental health, and personal agency Eliciting background narratives about who people are, where they are from, and how migration impacts at all levels of daily life, and consequently on their health and wellbeing, is integral to culturally safe practice with people with refugee backgrounds	Promoting community-level engagement with primary-care services may help to reduce inequalities for refugee populations Nurses to influence policy makers to recognise the individual, social, cultural and historical factors that affect the health of migrants Health services to engage with people with refugee backgrounds in culturally safe and responsive ways, through a diverse health workforce, both at policy development level and at on-the ground service level
Shrestha-Ranjit, Payne, Koziol-McLain, Crezee, and Manias (2020) Availability, Accessibility, Acceptability, and Quality of Interpreting Services to Refugee Women in New Zealand. *Qualitative Health Research*, *30*(11), 1697–1709 10.1177/1049732320924360	To examine effectiveness of interpreting services for refugee women in New Zealand	Qualitative	Purposive	32 Bhutanese women and eight Bhutanese men; 12 individual interviews with health professionals 18 to 82 years and men’s from 26 to 55 years	There are inadequacies and constraints in the provision of a socio-culturally and linguistically effective interpreting service to Bhutanese women	To establish community navigators to facilitate coordinated care that meet the sociocultural and gender-specific needs of Bhutanese refugees To develop health information resources in Nepali language and utilize them to enhance communication with Bhutanese refugees for effective primary health care services To advocate for refugee patients regarding their rights and responsibilities in their host nations
Shrestha-Ranjit, J. M., Patterson, E., Manias, E., Payne, D., & Koziol-McLain, J. (2017) Effectiveness of primary health care services in addressing mental health needs of minority refugee population in New Zealand. Issues in Mental Health Nursing, 38(4), 290–300. 10.1080/01612840.2017.1283375	To examine the effectiveness of primary health care services in addressing mental health needs of Bhutanese refugee women resettled in New Zealand	Exploratory Qualitative study Interviews and FGDs	Purposive	In all, 32 Bhutanese women participated with their ages ranging from 18 to 82 years eight Bhutanese men aged 26 to 55 years 12 individual interviews with health professionals	This study has reflected a diversity of viewpoints of service users Sources of mental distress were: Language difficulties Family separation Fragmented services Financial constraints Lack of spiritual and social support networks Language barrier compounded by lack of professional interpreter services Need for cultural awareness and education	Future research to explore Bhutanese refugee women’s experiences related to gender discrimination and its impact on their mental wellbeing after they resettled in New Zealand and other host countries Recommends to address inadequacies found in the findings
Akhtar, Heydon, and Norris (2021) Bringing Medicine from Pakistan and Self Medication Among Pakistani Mothers in New Zealand. *Journal of immigrant and minority health*, *24*(3), 682–688. 10.1007/s10903-021-01228-1	To explore the self-medication practices of Pakistani mothers for their children and their reasons for self-medication	Qualitative	Purposive	23 migrant women (Pakistan) aged 18yrs +	The requirement for a prescription and long waits and delay in GP appointments were the critical factors for self-medication in children. Themes were: Self-Medication for Their Children before taking to the doctor; Bringing Medicine from Pakistan in fear of not being able to manage the children’s illness Reasons for Self-Medication is medicine is heap and easily accessible without a prescription Types of Medicines used for self- medication were antipyretics, anti-allergic, analgesics (NSAIDs), eye and nasal drops, topical steroid creams, and Flagyl® for stomach problems	The Ministry of Health can develop healthcare awareness programs targeting new immigrants about antibiotic resistance and the potential risk of self-medication practice to prevent this self-medication practice and increase utilisation of health care
Henrickson, M., & Fisher, M. (2016) 'Treating Africans differently': using skin colour as proxy for HIV risk. *Journal of clinical nursing, 25(13–14)*, 1941–1949. 10.1111/jocn.13212	To investigate the issues of stigma and microaggressions and their effects on Black African communities	First study- qualitative Second study-Mixed method study	Purposive	First study, interviewed 13 Black Africans living with HIV Second study, surveyed 703 Black African new settlers, and included 131 participants in 23 different focus groups	Participants reported experiences of stigma and microaggressions based on their race, and a lack of knowledge about HIV in non-HIV specialist nurses and other health care workers Participants experienced poor health care and education practices, professional prejudice against colleagues living with HIV and institutional challenges including failure to protect patient confidentiality	Previous recommendations for increased and effective education and training in HIV have not been implemented
Cassim S et al. (2022) ‘Look, wait, I’ll translate’: refugee women’s experiences with interpreters in healthcare in Aotearoa New Zealand. *Australian Journal of Primary Health 28(4),* 296–302. 10.1071/PY21256	To explore refugee women's experiences of interpreters in healthcare in Aotearoa, New Zealand (NZ	Qualitative	Snow balling	Nine women aged between 20-50 years origin included: Eritrea, Afghanistan, Syria, Somalia, and Thailand	Patients asked to pay for interpreters Language discrepancies and different dialects Difficulties in making an appt without a GP Using family members as interpreters Breach of privacy	Achieving equitable healthcare for refugee women entails putting in place accessible and robust communicative infrastructure in NZ
Cassim, S., Ali, M., Kidd, J., Keenan, R., Begum, F., Jamil, D.,... Lawrenson, R. (2022). The experiences of refugee Muslim women in the Aotearoa New Zealand healthcare system. Kōtuitui: *New Zealand Journal of Social Sciences Online, 17(1),* 75–89. 10.1080/1177083X.2021.1947330	To explore the experiences of refugee Muslim women as they accessed and navigated the healthcare system in Aotearoa New Zealand	Qualitative	Snow balling	Nine Muslim women who arrived in NZ as refugees	Various structural barriers to accessing healthcare were identified such as cost and issues with interpreters, as well as instances of othering in the healthcare settings experienced by refugee Muslim women	To tackle inequity in the health system, structural and institutional barriers need to be addressed first, to prompt other levels of othering and discrimination to reduce over time
Jayan, P., & Dutta, M. J. (2021) Nobody cares about us: COVID-19 and voices of refugees from Aotearoa New Zealand. *Communication Research and Practice, 7(4),* 361–378. 10.1080/22041451.2021.1994686	To examine how the refugee communities navigated through the prevailing structural impediments to health during the pandemic	Qualitative	Snowballing	30 refugees (females and males) from Nepal, Afghanistan, Myanmar, Thailand and Bhutan	Lack of support services, inaccessibility of healthcare services and limitations in mobility	
Park, C., Loy, J. H., Lillis, S., & Menkes, D. B. (2022) What stops Korean immigrants from accessing child and adolescent mental health services 19. 10.1186/s13034-022-00455-0	To understand barriers to service access from Korean parents’ perspectives	Qualitative	Purposive	31 Korean parents of children aged 18 and under	Attitudinal barriers included attribution of mental illness to external stressors or parenting problems, social stigma, denial or normalization of children’s behaviour, fear of family disempowerment, and mistrust of public mental health services	Measures to improve access, for example by countering stigma, are urgently required
Akhtar, S. S., Heydon, S., & Norris, P. (2021). Access to the healthcare system: Experiences and perspectives of Pakistani immigrant mothers in New Zealand. *Journal of migration and health*, *5*, 100,077. 10.1016/j.jmh.2021.100077	To explore Pakistani immigrant mothers’ experiences and perspectives on navigating the healthcare system of a new country	Qualitative	Purposive	23 mothers in Wellington	Lack of knowledge, different expectations, and experiences of healthcare services inhibited their utilization of healthcare. Most mothers treated their children at home before visiting a general practitioner (GP) due to previous perceived unsatisfactory experiences, such as lack of availability of GP appointments for the same or next day, or long waiting times at emergency departments and after-hours medical facilities	Immigrant mothers need to feel they are getting the right services at the right time to ensure and promote better health outcomes. Identifying the barriers and promoting information about the healthcare system can play an essential role in the appropriate use of health services by immigrant mothers

Sample sizes of the studies ranged from nine to sixty participants. The length of stay of participants in Aotearoa prior to the studies also varied, with two studies not stating this information [35, 43]. Three studies had an average of four and five years for women and men, respectively [33, 34, 38], one study was between three months to 21 years [40], one study between six months to three years [26], two studies with an average of 3.25 years [41, 42] and another two studies between 1 and 19 years [36, 37]. Two of the studies did not include any information of length of stay as they focused on healthcare providers [5, 39].

The studies predominantly had women participants. Two studies focused on women only [36, 37], two studies were women with children [41, 42], and one study was with parents though women made up most of the participants [43]. Of the studies, four studies had a small proportion of men in the sample group [26, 33–35]. For instance, the smallest sample had eight males [33, 34]. Some studies did not mention the gender of participants, including one study on new settlers [40], two studies drew on healthcare providers [5, 39], and one study involved refugees and healthcare providers as participants [33].

### Barriers to Accessing Healthcare Services

**Fig. 2:**
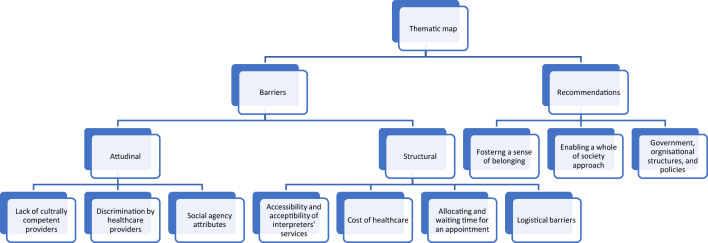
Thematic map

All studies indicated the existence of barriers in accessing healthcare amongst migrants and refugees due to several commonly overlapping factors. Three major themes were constructed: attitudinal barriers, structural barriers, and recommendations to improve access to healthcare in various settings (Fig. 2).

### Attitudinal Barriers

Attitudinal barriers are pervasive perceptions, beliefs, and value systems that societies, communities or specific individuals hold that in turn can influence healthcare access [44]. These attitudinal barriers were found to relate to the lack of culturally competent providers, discrimination by healthcare providers, and social agency attributes.

#### Lack of Culturally Competent Providers

The lack of healthcare providers’ sensitivity to the cultural backgrounds of migrant and refugee patients was a common theme in most studies [26, 33–35, 39, 43] [41] and considered as a justification for participants’ lack of trust and dissatisfaction in the health system. In particular for migrants, Park, Loy, Lillis and Menkes [43] and Akhtar, et al. [41] found participants sought health advise from their community groups or returned to their home country to seek health services. The finding on the lack of culturally competent healthcare providers was also echoed by providers themselves who reported not being adequately prepared to deal with the complex health issues of refugees, highlighting a perceived lack of preparedness of the health care delivery systems and workforce [5, 33, 39]. This included allocation of insufficient resources to general practices to allow refugees to access care at no cost, provision of interpretation services, and the lack of financial reimbursement to cater for extended consultations [39].

#### Discrimination by Healthcare Providers

Participants from migrant, refugee, and refugee-like backgrounds discussed experiences of ostracism in both primary and secondary health care encounters [5, 34, 36, 40, 42, 43]. For instance, Muslim women with refugee backgrounds felt they were underserved, such as being given painkillers without a thorough investigation of their symptoms [36], whilst Pakistani migrant women did not feel listened to or given no medication at all [42]. Another study on Black African migrants reported stigma related to race and a lack of provider knowledge of illness [40]. Peculiar to one study on Korean immigrants [43], participants perceived that they were stigmatised as having children with behavioural problems, which required mental health service involvement and subsequently led participants to avoid service referral and attempt to solve problems by themselves. Some primary care practices explained the perceived discrimination by refugees and refugee-like migrants, often observed to be the reluctance of health providers to enrol these populations due to the health providers’ perceived inherent complexity of their needs and potential costs [5, 34, 39], like developing relationships with allied service providers for triaging refugee patients for services and programmes that would meet their needs [39]. Coined as ‘charity’ and ‘unpaid’ work, the inherent complexity and potential costs were seen to place considerable burden on reception staff further limiting the functions of human resources and negatively impacting upon general practitioners’ (GPs) capacity to manage caseloads.

#### Social Agency Attributes

The review found personal, social and cultural attributes of migrant populations were seen to hinder healthcare access for women with former refugee status [26, 33, 34]. Women’s caring roles within their family, and later for their own children, largely shaped their future health seeking behaviours [26]. For instance, most female participants had had no opportunities prior to migration for formal education and were therefore illiterate and needed to rely on others, either their families (usually children) or friends for communicating their health needs [33]. Other gender-specific resettlement challenges were characterised by significant knowledge gaps about health and medical treatments evidenced by both migrant and refugee women participants further restricting their ability to follow the treatment courses [34, 41]. Moreover, these women participants never complained, or questioned the services they received [34].

### Structural Barriers

Structural barriers to accessing healthcare were reported more often than attitudinal barriers. Structural barriers are defined as the policies and frameworks that privilege specific community segments by regulating the accessibility of resources for others [45].

#### Accessibility and Acceptability of Interpreters’ Services

The major theme across most studies was language barriers and its effects on navigating, accessing, and utilising health services for migrants, refugees, and refugee-like migrants [5, 26, 33–39, 43]. This challenge was further exacerbated when some services were seen to have a lack of access to readily translated information on COVID-19 or support services as reported by Jayan and Dutta [35] for refugees. They also found that some healthcare institutions prohibited patients from bringing family or community members to support people with language difficulties and help to address anxiety regarding health procedures during COVID-19 lockdowns. Other participants with former refugee backgrounds reported a lack of professionally trained interpreters [33, 34], leading to language discrepancies for both migrants and refugees [37, 43]. There were also concerns when former refugees were offered interpreters of the opposite sex which was inappropriate from a cultural and/or religious perspective [26]. Similar barriers were found when participants of former refugee backgrounds and interpreters belonged to the same minority group resulting in participants not openly communicating their health needs due to the risk of breaching privacy by the interpreters [37]. One study found that refugee-like migrants believed in-person interpreters improved connection and understanding by having ‘little conversations’ outside the treatment room, giving results, and supporting with administrative processes [5].

#### Cost of Healthcare

Six studies described financial constraints as one of the key barriers to accessing healthcare [5, 33, 37, 39, 41, 42]. In the study by Akhtar, et al. [42], the cost to visit a GP was too expensive and seen as prohibitive as many of the migrant women relied on 'the government benefit' as their only source of income. Also in their view, after paying so much for an appointment, they came away without a prescription and feelings of discomfort and worry about not being adequately listened to. Some migrants therefore opted to bring medicines from their home country that were cheap and easily accessible [41]. For refugees, Cassim, et al. [37], found that GPs and after-hours services did not provide interpreter services which necessitated patients to bring their own interpreters at significant personal cost, which posed further barriers to accessing healthcare. However, some general practices enrolled in the Refugee Resettlement Programmes in Aotearoa could offer interpreter services at no cost [39]. However, the eligibility criteria excluded ‘refugee-like migrants’ for free interpreter services [5].

#### Allocated and Waiting Time for an Appointment

General practices operated under a business model that was seen to be in competing demand with the moral and ethical responsibilities of healthcare service delivery [39]. This was supported by reported experiences for both migrants and refugees of rushed appointments, long waiting periods for GP appointments, and having to use after-hours or emergency services [5, 33, 38, 39, 42]. GPs explained the consultation periods were only 15 min for everyone even when an interpreter was used [38], whilst other GPs, appointment times increased from the standard 15 min to 30 min when factoring in time for interpreting [5, 39]. The longer time required for consultations was perceived as a burden on the system by healthcare providers [39], yet providers still felt the allocated time was not suffient to address the complex healthcare needs presented by former refugees [38].

#### Lack of Information of the Health System

Lack of information about the health system and the inability to navigate through it was reported by both migrants and refugees [33, 35, 39, 42, 43]. For instance, migrants stated that it took them around two years to understand and navigate the health system in Aotearoa [42], with others reporting not being provided with information by their local GPs about services available [43]. In the case of former refugees, and specific to COVID-19 lockdowns, former refugees felt left out, with less access to support services such as help with completing forms for local government support [35]. Similarly, refugee health service providers also acknowledged their uncertainty about the services offered by other refugee health services providers, leading to apprehension about referrals to other services [39].

#### Logistical Barriers

Having knowledge about existing services was considered important, but often knowing how to access these services or who to contact was challenging for migrants and refugees [35, 42, 43]. For mothers who could not drive or did not have a car, using public transport to access healthcare was particularly difficult for those who had two or more children [42]. Mobility barriers were also reported during COVID-19 lockdowns where participants’ support services was disrupted [35].

### Recommendations to Improve Access to Health Care for Migrant and Refugees

Studies’ recommendations to improve services were grouped into three sub-themes, including: fostering a sense of belonging, enabling a whole-of-society approach, and government, organisational structures, and policies.

#### Fostering a Sense of Belonging

Former refugees require a sense of community or connectedness as a basic human need to maintain their identity, physical well-being, and mental health. A range of strategies to address those needs within the wider society for former refugees were outlined in some studies [26, 33, 34, 39]. These strategies included a better-structured resettlement support programme, on-the-job training, placements, and English language lessons. Important to one study was the recognition and utilisation of former refugees with healthcare skills to promote refugee health outcomes which in turn provides employment opportunities [34]. Other studies recommended that Aotearoa healthcare providers advocate for people from former refugee backgrounds to influence policy makers to recognise the unique individual, social, cultural and historical factors that affect their health and promote a culture of acceptance that celebrates diversity [26, 39].

A study on migrant health proposed enabling or supporting migrants’ health belief systems [43]. For instance, those who have been less integrated in their country of resettlement tended to adhere to health beliefs prevalent at the time they left their home country. This approach could, in the participants’ opinion, favour not only accommodating cultural practices in the provision of care, but also increasing their trust in service providers.

#### Enabling a Whole-of-Society Approach

A whole-of-society approach is a key concept that represents a broader approach beyond public authorities and relevant stakeholders, to engage individuals, families, communities, intergovernmental organisations, religious institutions, and so forth [46], to collaboratively work together to improve access to health care services amongst migrants and refugees. Bringing together all these players was seen as crucial to address the needs of former refugees and refugee-like migrants [5, 26, 33, 37, 39]. Some studies commended the integration of a gender perspective in the delivery of these services [36, 39], which was perceived to be “culturally appropriate practice, facilitating relationship building and trust, as well as helping with providing smoother pathways to and through general practice for refugee women patients” [39]. For instance, some refugee women patients prefer telephone or video interpreters, particularly for sensitive issues, such as sexual health or during physical examinations [36].

Some studies recommended that refugee health service providers collaborate with non-government organisations that work with refugees [5, 33, 39] to establish an interprofessional team within and across practices, an interconnecting network that shares information [5, 39], and invest in skill development and teamwork between practices [5]. For instance, refugee resettlement agencies could share best practices and information with health service providers on refugees and their complex health issues [33]. Additionally, promoting community-level engagement with primary care services may help to reduce inequalities for former refugee populations [34, 39]. This was supported by other studies who found the use of community/health navigators/intercultural mediators to be crucial cultural resources in navigating health and well-being journeys of refugees and communicating between healthcare providers and patients [26]. Similarly, other studies argued for refugee healthcare professionals and general care practises to work together to better meet the language development and health needs of people who resettle in Aotearoa [34–36, 39] [5]. This included a nationally coordinated network of trained healthcare interpreters whose services can be utilised in-person (based on location) or through video conferencing facilities.

#### Government, Organisational Structures, and Policies

The call for government and migrant and refugee healthcare organisational structures and processes to enable providers to address the core health needs of migrant, refugees, and refugee-like migrants were evident in many studies [5, 26, 34–37, 43, 47]. For refugee health, most studies highlighted the need for culturally centred and context-driven policies [26, 34, 35, 37–39, 43, 47]. This included contemporary evidence-based clinical guidelines in health settings, cultural sensitivity training, health literacy training, mentoring and other development opportunities for health and care professionals working with former refugee communities. Notably, Field, et al. [26] recommended a diverse health workforce at both the community service and policy development levels. Similarly, healthcare professionals who worked in culturally diverse contexts found practical knowledge acquired through experience with people from different cultures cultivated an understanding of ‘difference’ in terms of cultural and gender norms that they would then operationalise into their practice with refugees [39].

The implementation of policies that fund health services for former refugees was a reoccurring recommendation across several studies [5, 26, 34, 36–38]. Addressing structural barriers that compound the struggles of refugees to access quality and appropriate health services, such as time constraints during consultations [5, 34] and accessible and acceptable English language support services need to be addressed [5, 26, 34, 36–38]. Particular to interpretation services, authors proposed the allocation and provision of funding for interpreters equitably across primary and secondary healthcare services in a manner that suits the demographics of the populations they serve [36]. Other studies also pointed to the need for English courses for a longer period of time to help reduce language barriers [26, 34] and access to translated material about support services during public health emergencies like the COVID-19 pandemic [35].

## Discussion

This synthesis of findings from 13 studies found that most studies focused on refugees and highlighted the concerns of poorer health outcomes, mental health, general well-being, and social care needs of this group [48]. This review confirms a prior integrative review (28 studies) on current knowledge on the health of immigrants in Aotearoa, which found that studies on immigrant health mainly focused on refugee health [49], owing to their complex physical and mental health needs that are shaped by experienced in their country of origin and their migration journey. These experiences may increase vulnerabilites to chronic and infectious diseases. However, many migrants and migrants in irregular situation like refugee-like migrants are given a migrant status that limits their entitlements and access to health. Migrants and refugee-like migrants face high user fees, stigma, are treated by healthcare providers with inadequate cultural competency, and lack access to adequate interpretation services, in addition to having low levels of health literacy. The different legal frameworks that differeniatiate entitlements to healthcare between migrants and refugees imply the existence of inequities in the provsion of health services for migrants and refugees, thereby supporting calls to countries accepting migrants and refugees to incorporate the needs of migrants and refugess in national and local health policies, finance, planning, implementation and monitoring [50]. The same also applies for other refugee categories like asylum seekers and convention refugees [51].

The included studies used length of residence as an underpinning concept relating to healthcare access. The review demonstrated that access barriers were experienced despite the number of years settled in the host country [52]. Such experiences in accessing and utilising mainstream healthcare points towards ongoing systemic factors related to discrimination and marginalisation of migrants and refugees, where these groups are inequitably positioned within society in Aotearoa regardless of the years they have been resettled [53, 54].

We also noted most studies focused on women and children, indicating barriers to healthcare are greater than those for men; and their status as unserved members of society, yet the intersectionality of gender and age continue to be overlooked within the context of Aotearoa's recent health reforms for this population [55]. Issues behind inadequate access to healthcare echo many other urgent development issues, such as gender and socio-economic hierarchies that contribute to unequal distribution of power and resources, poverty and unemployment, and low literacy levels [56]. However, one study [42] demonstrated that educated migrant women also experience barriers in accessing healthcare, suggesting that not all barriers are a result of lack of education or literacy, but arise from structural social inequities and structural racism. While it is important to continue to give attention to women and children given their compounded marginalisation, efforts must also be put into understanding migrant and refugee men’s access to, and engagement with, health care services. Often masculine ideals increases gender inequalities to accessing healthcare, making men invisible within the healthcare system [57].

This review showed that when both migrants and refugees move to another country, they take with them their prior experience, cultural beliefs and practises, and knowledge [5, 26, 33, 39, 41–43]. For instance, participants with migrant backgrounds expressed dissatisfaction with their access to the healthcare system where healthcare professionals were not able to meet their cultural expectations when providing care [43]. The failure to meet the cultural expectations in addressing health needs were echoed in studies with healthcare providers as participants acknowledged their lack of awareness of the diversity and cultural appropriateness of specific speciality services of other healthcare providers [5, 33, 39]. This may indicate Aotearoa’s healthcare structure and processes being dominated by the specific cultural context, with little recognition of migrant and refugees’ beliefs about disease, treatment, and practices; implying health equalities in health services exist between the mainstream population and those of migrant and refugee communities.

The struggle with long waiting times to enrol with a GP and book an appointment was noted [5, 33, 39, 42], resulting in some migrants self-medicating with medicines from their home country [41]. Even where funding exists, practices had no capacity to take on higher caseloads, and some primary care practices are reluctant to enrol refugees and migrants as they are mandated to [5, 33, 39]. This may be due to primary care practices’ perceiving inherent complex physical, psychological, and social problems of refugees and consequences of lengthy consultation appointments for services run on a business model [23]. This has been previously reported in other studies as well [58, 59]. While one study found the reluctance to be on the grounds of discrimination based on their identities of ethnicites, countries of origin, and socio-economic status, thereby normalising inequities experienced by racialised communities [60], it should be noted that there is an ongoing struggle for patients to book health appointments nationwide due to an ageing GP workforce and not training enough GPs to replace those retiring [61]. Nonetheless, these barriers are thought to contribute to migrants and refugees accessing hospital emergency departments for general health issues rather than accessing GPs [62]. This additional strain further supports an overwhelming feeling of migrants and refugees being unable to access urgent healthcare services [63].

The reoccurring communication challenges brought about by language barriers also does not fully explain the lack of access to health services. Privacy breaches when utilising professional interpreters, particularly in small towns where interpreters are often limited and are well known to the community, often results in people being reluctant to share information about their personal health conditions during appointments [64]. While many district health boards in Aotearoa (which were disestablished and merged into Health New Zealand as of 1 July 2022 as part of the national health reforms) now have policies on interpreter use, these are far from consistently implemented (see work by Gray, et al. [65]). Migrant and refugee patients are generally unaware of the Health and Disability Code of Patient Rights and the free provision of interpreters. This may suggest that migrant and refugee patients may not exercise their right to an interpreter if they are not aware of their rights. They therefore do not request for interpreting services and the providers may assume that they do not need it because they did not ask. It may also be that healthcare providers are selective with who is offered interpretation services. Further, the right for New Zealanders to sue doctors for alleged medical treatment injury was removed, reducing the pressures to enforce the provision of interpretation services [66].

One systematic review through healthcare providers’ lens (37 studies) found providers addressed policies and frameworks that regulated the accessibility to health care by migrants by somewhat ignoring their migrant status, and using various strategies, including seeking help from civil society groups, to support their clinical practice [51]. The incorporation of transnational insights for contemporary health care based primarily on migrant and refugee community perspectives, though not yet incorporated as a critical component of health service research, promises to be useful in provider-patient encounters to address inequities in accessing healthcare as reported elsewhere [67]. Further, a comparison study with Aotearoa found the use of patient navigators not only provided adequate support and resources for migrants and refugees, but also created a financial return [68]. Every dollar invested in patient navigators saved about $6, and no-show rates dropped to 54% saving $35,000 [68]. Equally important was the provision and access to education and information dissemination among migrant communities in order to navigate the health system [43].

The review demonstrates that the health and wellbeing of migrants and refugees receives minimal attention because of the lack of relevant national policies in Aotearoa, Australia, and Canada [3, 49, 55]. This notion was amplified during the COVID-19 pandemic as seen in other international studies [69, 70], and is further reinforced when strategies formulated in the current national health reform do not clearly demonstrate how migrant and refugee health needs will be met [55]. This is often due to the invisibility and voicelessness of migrants and refugees in dominant discourse [49]. The invisibility of migrants and refugees often makes integration in society difficult thereby impacting on several factors including challenges with securing employment, directly contributing to low/poor economic status, and inability to afford health care services as Akhtar, et al. [42] has shown. This is particularly true for those with limited English proficiency [71, 72]. Although contrasting evidence shows that diversity is respected and accommodated in Aotearoa, it is far from being promoted [73]. Emphasis tends to be on integration; that is addressing settlement issues and encouraging the learning of the host-country language and culture [73]. This is not a counter to integration, but to allow migrants and refugees to maintain their transnational identity, and preserve, honour and respect their cultural, religious, and linguistic ties, Salahshour [73] proposes increased policies and initiative encouraging better intercultural understanding of other minority groups while maintaining the attention given to Māori and Pacific people as being mutually exclusive. Allowing migrants and refugees to maintain their transnational identity and among other identities may provide an alternate and viable avenue to cultivate a sense of belonging, contribute to social and economic capital, and result in the health benefits for migrant and refugee communities as seen elsewhere [74, 75].

### Strengths and Limitations

This is the first review that synthesises the barriers to accessing healthcare among migrants and refugees, and recommendations for improvement within Aotearoa. The review included relatively few studies focusing on healthcare providers perspectives, which would have provided rich context-specific findings to complement migrants’ and refugees’ perspectives. The search of eligible studies was restricted to nine research databases which may have missed studies meeting the inclusion criteria. However, we did search references lists for additional studies. Our review did not include studies published in languages other than English so we may have missed some relevant studies. By only including empirical, peer-reviewed studies, we also may have missed potentially relevant grey literature published by government agencies, non-governmental agencies, or private foundations. Being an integrative review, we did not assess for quality; thus, future research could conduct a systematic review to understand the quality of research in this field.

## Conclusion

This review found varying attitudinal and structural barriers hindered equitable access to healthcare for migrants and refugees in Aotearoa. Ongoing barriers in the domains of funding policies, such as interpreter services and time allocated for consultations, presented challenges to healthcare providers to meet the health needs of their patients with migrant or refugee backgrounds. The review highlighted recommendations for improvement, such as having a nationally coordinated network of organisations to foster collaboration among stakeholders and promoting a sense of belonging of migrant and refugee communities. The health of migrants and refugees may be determined by their ability to be included in communities and access appropriate and acceptable health services in their host country. In summary, addressing the root causes of the identified barriers to equitable access to health care for migrant and refugee populations in Aotearoa is complex. Policy makers need to recognise the multitude of factors that affect the health of migrants and refugees and promote a conducive environment through culturally sensitive and responsive policies, that promote a multi-cultural health workforce, address discriminatory workforce practices, and provide culturally and linguistically appropriate primary care services and health education to enable healthcare providers to meet the needs of these diverse populations.
